# NR2F2 Orphan Nuclear Receptor is Involved in Estrogen Receptor Alpha-Mediated Transcriptional Regulation in Luminal A Breast Cancer Cells

**DOI:** 10.3390/ijms21061910

**Published:** 2020-03-11

**Authors:** Edina Erdős, Bálint László Bálint

**Affiliations:** 1 Genomic Medicine and Bioinformatic Core Facility, Department of Biochemistry and Molecular Biology, Faculty of Medicine, University of Debrecen, 98 Nagyerdei krt., H-4032 Debrecen, Hungary; edina.erdos@med.unideb.hu; 2 Doctoral School of Molecular Cell and Immune Biology, Faculty of Medicine, University of Debrecen, 98 Nagyerdei krt., H-4032 Debrecen, Hungary

**Keywords:** estrogen receptor alpha, NR2F2, cistrome, active histone modifications, chromatin interactions, breast cancer, luminal A subtype

## Abstract

Nuclear Receptor Subfamily 2 Group F Member 2 (NR2F2) is a member of the steroid/thyroid hormone receptor superfamily with a crucial role in organogenesis, angiogenesis, cardiovascular development and tumorigenesis. However, there is limited knowledge about the cistrome and transcriptome of NR2F2 in breast cancer. In this study, we mapped the regulatory mechanism by NR2F2 using functional genomic methods. To investigate the clinical significance of NR2F2 in breast cancer, The Cancer Genome Atlas (TCGA) data were used. These results show that a high *NR2F2* is associated with better survival of a specific subset of patients, namely those with luminal A breast cancer. Therefore, genome-wide NR2F2 and estrogen receptor alpha (ERα) binding sites were mapped in luminal A breast cancer cells using chromatin immunoprecipitation followed by high-throughput sequencing (ChIP-Seq), revealing that most NR2F2 overlap with ERα that are co-occupied by forkhead box A1 (FOXA1) and GATA binding protein 3 (GATA3) in active enhancer regions. NR2F2 overlaps with highly frequent ERα chromatin interactions, which are essential for the formation of ERα-bound super-enhancers. In the process of the transcriptome profiling of NR2F2-depleted breast cancer cells such differentially expressed genes have been identified that are involved in endocrine therapy resistance and are also ERα target genes. Overall, these findings demonstrate that the NR2F2 nuclear receptor has a key role in ERα-mediated transcription and it can offer a potential therapeutic target in patients with luminal A breast cancer.

## 1. Introduction

Breast cancer is the most frequently diagnosed form of cancer among women in the world (GLOBOCAN 2018) [1]; its mortality, however, has decreased in recent years due to advances in available therapies. The presence of estrogen receptor alpha (ERα) in cancerous cells has a crucial role in the treatments, which associates with better survival and is the key target of endocrine therapy. ERα, as a nuclear receptor, binds with DNA-regulating elements due to the effect of ligand, which changes the activity of particular genes [2]. A large number of co-regulators have been identified in ERα regulatory complex [3]. These co-regulators can modulate the functional activity of ERα and offer potential therapeutic targets.

The chicken ovalbumin upstream promoter transcription factor II (COUP-TFII, otherwise known as NR2F2) belongs to the family of steroid/thyroid nuclear receptors [4]. Nuclear receptors regulate the expression of their target genes either negatively or positively due to the effect of the ligand. The natural ligand of NR2F2, however, has not yet been identified, thus, it is considered an orphan nuclear receptor. As a nuclear receptor, it can bind to the direct repeat of the AGGTCA motif both in homo- and heterodimer form with another nuclear receptor such as retinoid X receptor (RXR). NR2F2 binding is very flexible to nuclear receptor motifs, and it can recognize the motif of other nuclear receptors (ERα, hepatocyte nuclear factor 4 alpha (HNF4A), thyroid receptor (TR), vitamin D receptor (VDR)); thus, it results in a competition for DNA binding sites between nuclear receptors [5]. NR2F2 plays a role in such developmental processes as organogenesis, angiogenesis and lymphogenesis, and at the same time it has also been found to have a critical role in tumorigenesis [6]. It has been shown in breast cancer that the expression of the NR2F2 is lower in ER-negative cancer and it participates in chemoresistance in cell-type and agent-specific form [7]. Riggs et al. reported that a decreased NR2F2 expression correlates with antiestrogen resistance, i.e., if NR2F2 was knocked out of tamoxifen-sensitive MCF-7 cells, the effect of tamoxifen ended, but if NR2F2 was made to overexpress in tamoxifen-resistant cells, sensitivity to tamoxifen was reestablished [8]. All this supports that NR2F2 plays an important role in the response given to the treatment of breast cancer patients; however, the genome-wide regulation of NR2F2 is not known in breast cancer.

Several studies have reported that NR2F2 is a critical nuclear receptor in cardiovascular development [9,10,11,12], but we demonstrate that this nuclear receptor has a different molecular function in breast cancer. In this paper, we investigated the gene expression level of NR2F2 in patients with different breast cancer subtypes. NR2F2 is associated with good survival and prognosis in patients with ER-positive breast cancer. To understand the molecular mechanism of NR2F2 in breast cancer, we determined the cistrome and transcriptome of NR2F2 in the ER-positive breast cancer cell lines (MCF-7 and T47D) using the chromatin immunoprecipitation followed by high-throughput sequencing (ChIP-seq) and RNA-seq methods. This work provides evidence that NR2F2 is associated with ERα-mediated transcriptional programs. Our findings suggest the importance of NR2F2 in breast cancer treatment and prognosis.

## 2. Results

### 2.1. A High NR2F2 Expression Level Is Associated with Better Outcome in Patients with Luminal a Breast Cancer

**Figure 1:**
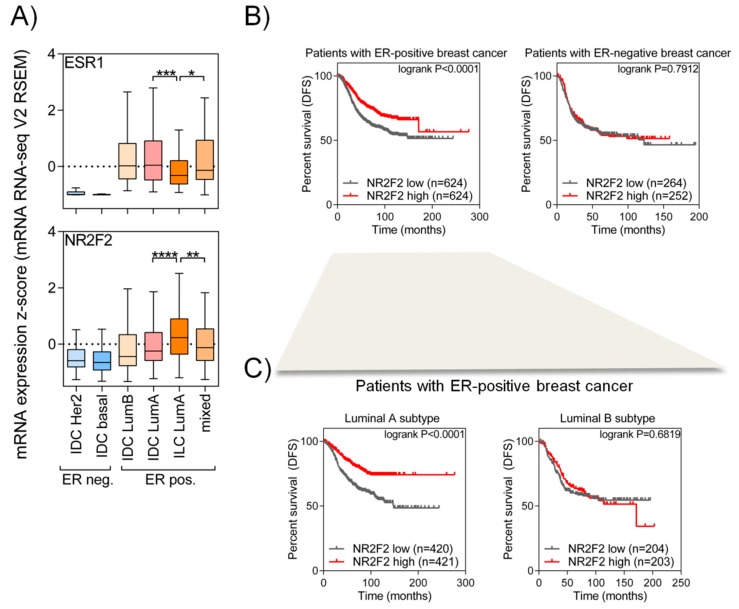
NR2F2 shows a high expression level in patients with ERα positive breast cancer. (**A**) Box plots show differences in *ESR1* (encoding ERα) (upper panel) and *NR2F2* (lower panel) gene expression between each subtype of breast cancer patients. Mann-Whitney test, * significant at *p* < 0.05, ** *p* <0.01, *** *p* <0.001, **** *p* <0.0001 values. (**B**) Kaplan-Meier analysis shows the disease-free survival of patients with ERα positive and ERα negative breast cancer based on high or low *NR2F2* expression. Mantel-Cox test was used. (**C**) Kaplan-Meier analysis shows the disease-free survival of patients with luminal A and luminal B breast cancer based on high or low *NR2F2* expression. A Mantel-Cox test was used.

To examine the expression of the *NR2F2* gene in different breast cancer subgroups, we used the RNA-Seq data of 817 patients derived from a TCGA database. Patients were divided into histologically invasive ductal carcinoma (IDC), invasive lobular carcinoma (ILC) and mixed IDC/ILC groups. The IDC patients were further divided into the PAM50 subtype, while ILC and mixed patients mostly exhibit luminal A subtype (90%); thus, there was no subcategorization in their case. In the six groups created this way, we investigated the expression of *ESR1* and *NR2F2* genes (Figure 1A). Patients with ER-negative breast cancer (IDC HER2+ and basal) show a low ESR1 and NR2F2 mRNA level. ER-positive breast cancer patients with luminal A and luminal B subtypes show a higher expression of *ESR1* and *NR2F2*. The expression of *NR2F2* is significantly the highest in ILC luminal A subtype compared to other groups (*p* < 0.01, Mann Whitney test).

To investigate the effects of *NR2F2* gene expression on survival, we first compared the survival of patients in ERα positive and negative sub-groups with the low and high expression of *NR2F2* (Figure 1B). We have found that patients with ER-positive breast cancer show significant (logrank *p* < 0.0001, Mantel-Cox test) differences in disease-free survival (DFS) based on *NR2F2* expression. Next, we investigated the DFS in two sub-groups (Luminal A and B) of patients with ER-positive breast cancer, disregarding the origin of cancerous cells (ductal or lobular) (Figure 1C). We have found that patients with luminal A breast cancer and a high expression of NR2F2 have better disease-free survival (logrank *p* < 0.0001, Mantel-Cox test) than those with a low *NR2F2* level. Patients with luminal B breast cancer show no difference in DFS. All these findings suggested that NR2F2 has an important role in ER-positive luminal A type breast cancer.

### 2.2. NR2F2 Overlaps with ERα Binding Events in Luminal A Breast Cancer Cells

**Figure 2:**
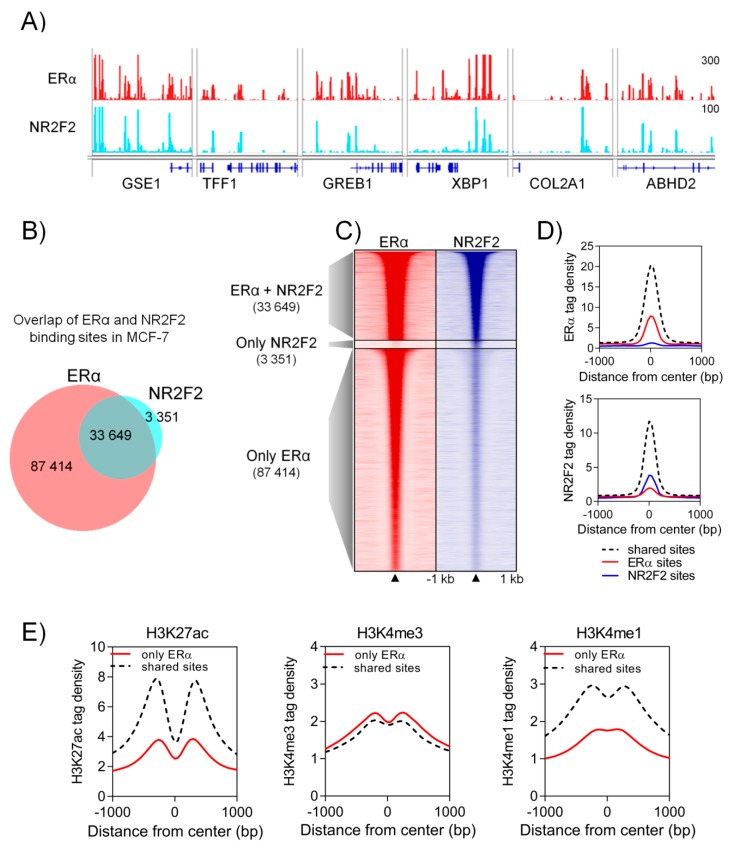
NR2F2 at ERα binding events in MCF-7 cells. (**A**) The Integrative Genomics Viewer IGV screenshot shows ERα and NR2F2 binding sites at ERα target genes. (**B**) Proportional Venn diagram and (**C**) heatmap represent the overlapping regions between ERα and NR2F2 binding sites. (**D**) Histograms show the ERα and NR2F2 tag density at shared and individual binding sites. (**E**) Histograms show the H3K27ac, H3K4me3 and H3K4me1 tag density at shared and individual ERα binding sites.

To investigate the cistrome of NR2F2 and its presence in the ERα-mediated transcriptional complex, we performed chromatin immunoprecipitation followed by sequencing (ChIP-seq) with NR2F2 and ERα antibodies using two luminal A breast cancer cells (MCF-7 and T47D). Two biological replicates were sequenced and then merged before peak calling. MCF-7 cells are cancerous and contain copy number variations that are overrepresented or underrepresented during peak calling. HMCan, a program for peak calling uses normalization methods for copy number variations [13]. Thus, we detected 38,107 NR2F2 binding sites and 121,097 ERα binding sites in MCF-7 cells using HMCan. ERα and NR2F2 peaks can be found at well-known ERα target genes such as *TFF1*, *GREB1* and *XBP1* (Figure 2A). To determine the number of overlapping regions between ERα and NR2F2, we used diffBind analysis. This analysis reveals that 90% of NR2F2 binding sites overlap with ERα (Figure 2B,C). We investigated the ERα and NR2F2 ChIP-seq signal intensities at the individual and shared ERα and NR2F2 binding sites. We found higher ERα and NR2F2 ChIP-seq signal intensities at shared ERα and NR2F2 binding sites than at individual ERα or NR2F2 binding sites (Figure 2D). These results were confirmed using T47D cells despite the very low number of NR2F2 binding sites in T47D (Figure S1). To define the specific regulatory elements (such as enhancers or promoters) at shared ERα and NR2F2 binding sites, we used a ChIP-seq dataset for activating histone modifications. This analysis reveals that shared ERα and NR2F2 binding sites show higher ChIP-seq intensities for active enhancer specific histone modifications (H3K27ac and H3K4me1) than the individual ERα binding sites (Figure 2E). Altogether, the vast majority of NR2F2 overlap with highly enriched ERα binding at active enhancer regions.

### 2.3. NR2F2 Binds to ERα Binding Sites Co-Occupied by FOXA1 and GATA3 Co-Regulators

**Figure 3:**
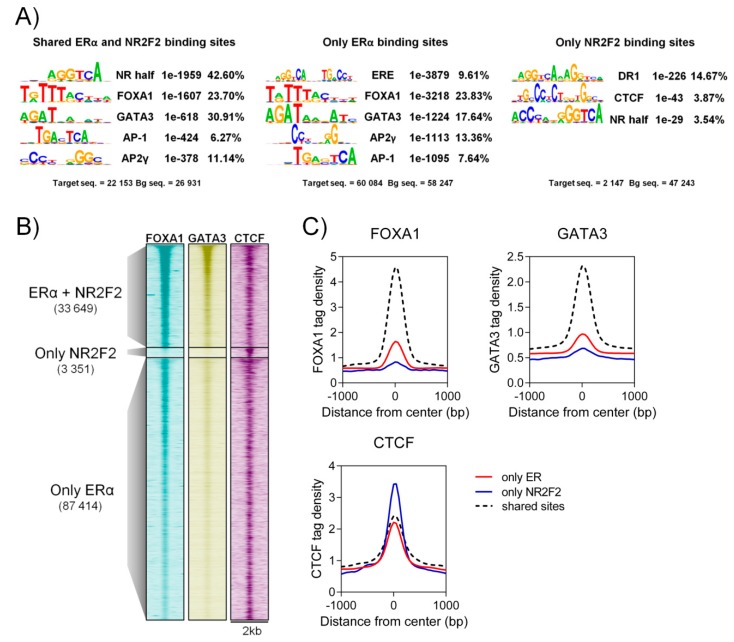
Co-factors at shared ERα and NR2F2 and individual binding sites in MCF-7. (**A**) Motif enrichment analysis at shared and individual ERα and NR2F2 binding sites. Motif logo, motif name, *p*-value and percent of target are represented. (**B**) Heatmap and (**C**) histograms represent the ChIP-seq signal intensities of FOXA1, GATA3 and CTCF at shared and individual binding sites in MCF-7 cells.

Based on earlier studies, many well-known co-regulators such as forkhead box A1 (FOXA1), GATA binding protein 3 (GATA3) and activator protein 2 gamma (AP2γ) have been identified at the ERα regulatory complex [14,15,16,17,18]. To investigate the co-binding of other transcription factors at individual and shared NR2F2 and ERα binding sites, we performed motif-enrichment analysis using Hypergeometric Optimization of Motif EnRichment (HOMER). Shared ERα and NR2F2 binding sites and individual ERα binding sites show the enrichment of nuclear receptor specific half-sites, FOXA1, GATA3, activator protein 1 (AP-1) and AP2γ motifs (Figure 3A). The estrogen response element (ERE) motif was enriched at individual ERα binding sites. Only the percentage of the GATA3 motif shows differences between individual and shared ERα binding sites (17.64% at only ERα vs. 30.91% at shared ERα). Direct repeat 1 (NR2F2 consensus sequences) and CTCF motif were enriched at individual NR2F2 binding sites. Based on this result, a well-known ERα co-factor FOXA1, GATA3 and CTCF binding intensities were investigated at individual and shared ERα and NR2F2 binding sites. We found higher FOXA1 and GATA3 ChIP-seq signal intensities at shared ERαα and NR2F2 binding sites than individual binding sites, while CTCF shows higher binding intensities at individual NR2F2 binding sites. Overall, our results suggest that NR2F2 binds to the ERα regulatory complex in the presence of FOXA1 and GATA3 co-factors.

### 2.4. NR2F2 Contributes to the Formation of ERα Super-Enhancers

**Figure 4:**
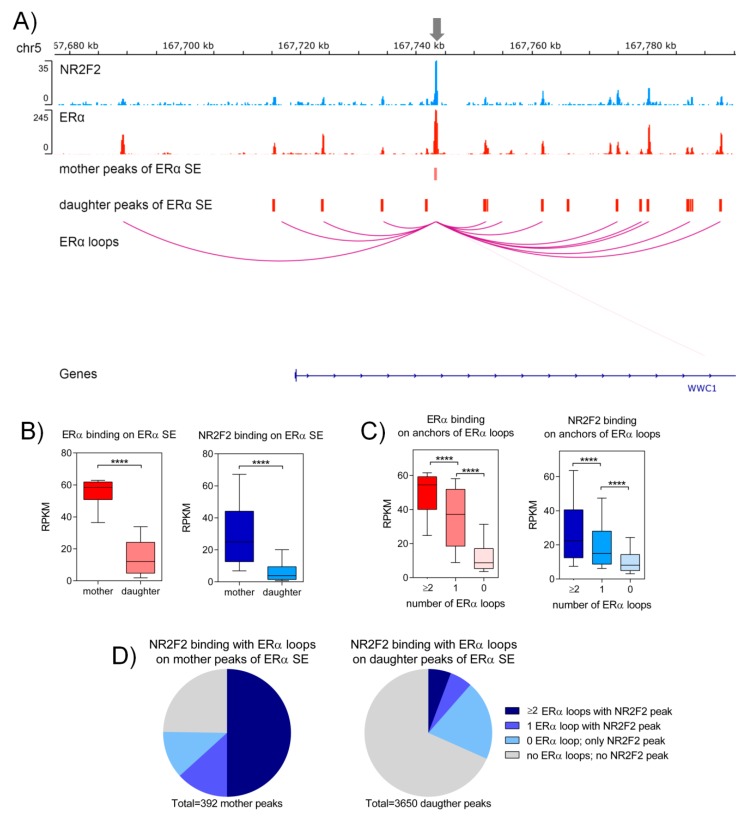
NR2F2 binds to mother regions of ERα super-enhancers (SE) in MCF-7 breast cancer cells. (**A**) IGV screenshot shows that NR2F2 and ERα binding sites marked the mother and daughter regions of ERα SE with ERα bound chromatin interactions at *WWC1* gene. (**B**) Box plots show the ERα and NR2F2 binding intensities on mother and daughter regions of ERα SE. Mann-Whitney test, **** significant at *p* < 0.0001. (**C**) Box plots show the ERα and NR2F2 binding intensities on anchor regions of ERα bound chromatin interactions based on number of interactions. Mann-Whitney test, **** significant at *p* < 0.0001. (**D**) Pie charts represent the percentage of mother (left) or daughter (right) regions of ERα SE overlapping with NR2F2 binding sites associated to ERα bound chromatin interactions.

Super-enhancers (SEs) are a large group of enhancers that are close to each other physically and correlate with high density binding of transcription factors and active chromatin regions (H3K27ac). [19]. Super-enhancers regulate genes that exhibit cell-type-specific regulation, contributing to the maintenance of cell identity, be it normal or cancerous [20]. The regulatory elements (enhancers, super-enhancers) are often located far away from the promoter of target genes. The three-dimensional (3D) structure and the organization of the genome make it possible for them to get closer to each other [21]. In our research group, we identified ERα-bound super-enhancers where mother and daughter regions of super-enhancers form a regulatory unit [22]. Here, we investigated the presence of NR2F2 within these ERα-bound super-enhancers. We have identified NR2F2 binding within ERα-bound super-enhancers in regions that are close to *WWC1*, *JARID2* and *SIAH2* genes (Figure 4A and Figure S2). Comparing the tag densities of ERα and NR2F2 ChIP-seq at mother and daughter regions of ERα-bound SEs revealed significantly higher ERα and NR2F2 binding (*p* < 0.0001 with Mann-Whitney test) on mother regions of ERα-bound SEs than on daughter regions of ERα-bound SEs (Figure 4B). Super-enhancers associate with high-frequency chromatin interactions [23,24,25]. To investigate the NR2F2 binding intensities at ERα-mediated long-range chromatin interactions, we used ERα ChIA-PET data from MCF-7 cells processed by ENCODE. We subdivided the NR2F2 and ERα binding sites into three groups (≥2, 1 or 0 loops) based on overlaps with numbers of ERα chromatin interactions (loops). Both ERα and NR2F2 with high-frequency ERα-bound loops showed significantly higher binding intensities (*p* < 0.0001 with Mann-Whitney test) than binding sites with one interaction or without interaction (Figure 4C). To gain insights into the role of NR2F2 at ERα-bound super-enhancers, we compared the ratio of mother and daughter regions of ERα-bound SEs with NR2F2 binding sites and ERα mediated interactions. Our results showed that the mother regions overlap to a greater extent with the NR2F2 binding sites correlated with ERα-mediated chromatin interactions than the daughter regions (63.2% of mother regions vs. 11.44% of daughter regions) (Figure 4D). Overall, our results demonstrate that NR2F2 with high-frequency ERα-mediated chromatin interactions is enriched mostly at mother regions of ERα-bound SEs to become a major contributor to the formation of ERα-bound super-enhancers.

### 2.5. NR2F2 Is Involved in ERα-Mediated Gene Expression in Breast Cancer Cells

**Figure 5:**
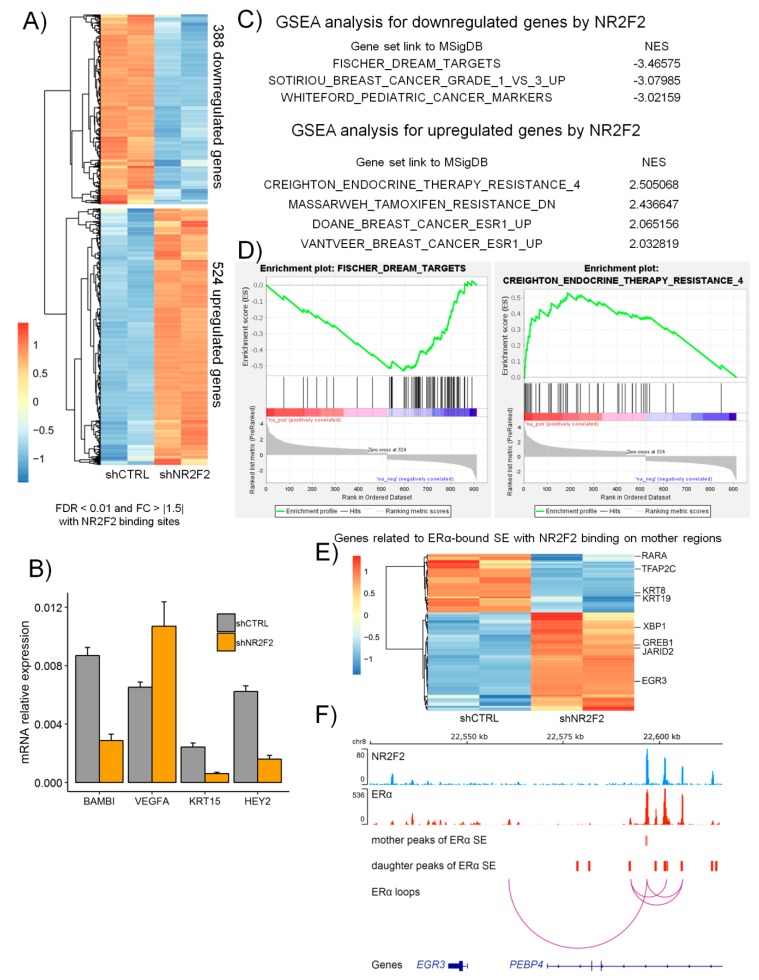
NR2F2 regulated transcriptional programs in breast cancer cells with luminal A subtype. (**A**) RNA-seq heatmap shows the clusters of differentially expressed genes in NR2F2 silenced MCF-7 cells using specific shRNA (shNR2F2). (**B**) RT-qPCR validation shows specific differentially expressed genes in MCF-7 cells. (**C**) Gene set enrichment analysis (GSEA) shows the gene sets that were significantly enriched in NR2F2 silenced MCF-7 cells. NES, normalized enrichment score. (**D**) GSEA plots for specific gene sets. (**E**) RNA-seq heatmap shows the expression levels of genes related to ERα SE with NR2F2 binding on mother regions in control (shCTRL) and shNR2F2 treated MCF-7 cells. (**F**) IGV screenshot shows that NR2F2 and ERα binding sites marked the mother and daughter regions of ERα SE with ERα bound loops at the *EGR3* gene.

To examine the regulation of gene expression by NR2F2 in luminal A breast cancer cells, we depleted NR2F2 using the lentiviral shRNA approach and then performed mRNA sequencing. After shRNA treatment, the level of NR2F2 mRNA was decreased in MCF-7 and T47D cells measured by RT-qPCR (Figure S3). We identified the differentially expressed genes in NR2F2 depleted MCF-7 cells compared to the control (shCTRL) treated MCF-7 cells using edgeR. We used those genes that are associated with NR2F2 binding sites based on ChIP-seq data. Thus, we identified 388 downregulated and 524 upregulated genes using statistical significance at the fold change (FC) >|1.5| and the false discovery rate (FDR) <0.01 (Figure 5A). The expression level of some genes such as *BAMBI*, *VEGFA*, *KRT15* and *HEY2* were confirmed by RT-qPCR (Figure 5B). Next, Gene Set Enrichment Analysis (GSEA) was performed (Figure 5C,D). Downregulated genes after NR2F2 depletion are associated with DREAM (dimerization partner, RB-like, E2F and multi-vulval class B) complex target genes [26]. DREAM plays an important role in the regulation of cell cycle [26]. Upregulated genes in NR2F2-depleted MCF-7 cells are similar to genes upregulated by estradiol and downregulated in endocrine resistance cells [27,28,29,30]. To investigate the expression level of genes related to ERα-bound super-enhancers in control and shNR2F2 treated cells, we found that almost half of the genes (99/236 genes expressed differentially) involved well-known ERα target genes such as *KRT8*, *XBP1*, *GREB1* and *EGR3* changed after NR2F2 depletion in MCF-7 cells (Figure 5E-F). Overall, these findings confirm the role of NR2F2 in ERα-mediated transcriptional regulations in breast cancer cells with luminal A subtype.

## 3. Discussion

Numerous studies have examined the role of NR2F2 in breast cancer cells and patients; however, its function has still not been clarified. In our paper, we demonstrated that breast cancer patients with the luminal A subtype who have a high NR2F2 expression show better survival. Here, we examined the regulatory mechanisms by NR2F2 in luminal A breast cancer cells on the genome level to investigate its role in the ERα regulatory complex.

Our results suggest that the high level of NR2F2 expression shows better survival in luminal A patients, and within that, ILC patients show a higher NR2F2 expression level than IDC patients. In the two carcinomas with a different histological origin, the survival of patients is very similar; however, the response of ILC patients to the therapy differs, despite the fact that both are ER-positive breast cancers [31]. This suggests that NR2F2 forms such an interaction with ERα that affects the therapeutic response.

There is increasing evidence that NR2F2 plays some kind of a role in the progression of breast cancer. Nagasaki et al. have shown that of the 119 breast cancer patients, 59% were NR2F2 positive for immunohistochemical staining, and the positive cases correlate with a bad clinical outcome and ER-positive status [32]. However, Zhang has found the opposite, claiming that the high expression of NR2F2 shows better overall and disease-free survival in breast cancer patients [7]. To clarify this issue, we performed a Kaplan-Meier analysis using a database collecting the gene expression and survival data of thousands of breast cancer patients [33]. Our results correlate with those of Zhang, in that the high expression of NR2F2 shows better survival; however, we have found that this is true only in ER-positive breast cancer patients, and in the case of ER-negative patients there is no difference in survival between the high and low expressions of NR2F2.

NR2F2, as a nuclear receptor, affects the transcription activity of numerous genes; however, its genome-wide distribution is not known in cancerous cells. Here, we mapped the NR2F2 binding sites genome-wide, using ChIP-seq data derived from luminal A breast cancer cells. The vast majority of NR2F2 binding sites show overlaps with ERα binding sites correlated with histone modifications (H3K27ac and H3K4me1), which are specific markers for the active enhancer regions. In our previous study, a greater extent of overlap has been demonstrated between NR2F2 and ERα binding sites [34]. Mohammed et al. have identified various ERα-associated proteins, including NR2F2, with the rapid immunoprecipitation mass spectrometry of endogenous proteins RIME method [35]. Previous studies have shown that some ERα co-factors such as FOXA1 and GATA3 act as pioneer factors in facilitating the ERα binding to chromatin [16,18]. Thus, these co-factors play an essential role in the ERα-mediated regulatory complex. Here, we showed that NR2F2 is co-occupied with ERα and its co-factors (FOXA1 and GATA3). These findings were confirmed by a recent study [36]. Rosenfeld et al. have identified that other nuclear receptors also bind to ERα MegaTrans complexes in a ligand-dependent manner via protein-protein interaction. For example, estrogen treatment causes interactions with RARα or RARγ [37], while adding estrogen to dexamethasone, a glucocorticoid receptor (GR) agonist, replaces RAR with sumoylated GR [38]. Sumoylated GR binds a co-repressor complex, thereby inhibiting ERα-dependent gene expression and enhancer activity [38]. Other studies have described interactions between other nuclear receptors such as PR, AR and LRH-1, capable of interacting with ERα in breast cancer [39,40,41,42]. We suppose a similar interaction between ERα and NR2F2 stimulated by a different signaling pathway. However, in the ChIP-Seq method, due to the high cell number, it is difficult to specify exactly which are the protein-protein interactions or competitions for nuclear receptor binding sites.

Super-enhancers represent a highly-organized transcriptional unit in gene expression [19]. The super-enhancers are correlated with a high density of transcription factors [20]. Here, we show that NR2F2 co-binds to high enriched ERα, FOXA1 and GATA3 binding sites. This result assumes the formation of a regulatory complex similar to super-enhancers. Based on previous results of our group, it means that mother and daughter enhancer regions play an important role in ERα–bound super-enhancer formation [22]. ERα at mother regions was pre-recruited before estradiol treatment. After estradiol treatment around the mother regions, ERα binds to daughter regions of super-enhancers. Therefore, we investigated the presence of NR2F2 at these super-enhancer regions. Our results demonstrate that NR2F2 with high-frequency ERα-mediated chromatin interactions binds to mother regions of ERα-bound super-enhancers without any treatment. Altogether, NR2F2 can contribute to the formation of ERα-bound super-enhancers.

Finally, the transcriptome profiling of NR2F2 depleted ER-positive breast cancer cells showed that NR2F2 plays a role in the expression of genes regulating cell cycle and that of estrogen responsive genes. Jiang et al. also found that NR2F2 silencing alters the expression of genes involved in the cell cycle [36]. Furthermore, Nakshatri et al. reported that NR2F2 plays an important role in cell cycle regulation in certain breast cancer cells by delaying the transition between late S and G2/M via regulation of cdk2 and cyclin D1 [43]. We investigated the changes in the expression of genes related to ERα-bound super-enhancers. This result confirmed that NR2F2 regulates the expression of the ERα target genes. In summary, these findings suggest a functional role of NR2F2 in ERα-mediated gene expression.

In our study, we performed NR2F2 and ERα ChIP-Seq from luminal A breast cancer cell lines. To investigate the effect of NR2F2 on gene expression, we performed RNA-Seq from NR2F2-depleted breast cancer cells. Based on these methods, our results showed that NR2F2 is present in ERα-mediated transcriptional regulation. Our findings suppose two mechanisms of interaction between these nuclear receptors: 1) protein-protein interactions within the same regulatory complex or 2) NR2F2 can compete for ERα binding sites. Further investigations are needed to study the contribution of direct NR2F2:DNA binding to the modulation of ERα-dependent transcription in breast cancer cells, such as generating breast cancer cells with mutant DNA-binding domain of NR2F2 and the assessment of the transcriptional effect of such NR2F2 mutants.

## 4. Materials and Methods

### 4.1. Cell Culture, Treatment

MCF-7 cells obtained from ECACC were cultured in DMEM media supplemented with 10% FBS, 1% penicillin-streptomycin in a 5% CO_2_ incubator. T47D cells were cultured in RPMI-1640 media supplemented with 10% FBS, 1% penicillin-streptomycin in a 5% CO2 incubator. The cells were hormone stripped for 3 days in phenol-free media with 10% charcoal-stripped FBS and 1% penicillin-streptomycin before treatment. After hormone deprivation, the cells were treated with 100 nM 17β-estradiol or 1 µM tamoxifen for 1 h. Absolute ethanol was used as control vehicle.

### 4.2. Gene Silencing

NR2F2 silenced MCF-7 and T47D cells were produced using Sigma MISSION^®^ Lentiviral Transduction Particles (NM_000125, TRCN0000003300) and Sigma MISSION^®^ pLKO.1-puro Non-Target shRNA Control Lentiviral Transduction Particles (SHC016V-1EA) according to the manufacturer’s instructions. Briefly, 2.5 × 10^5^ cells were transduced with the lentiviral particles at a MOI = 5, with 5 μg/mL polybrene, and then, were selected with 2 μg/mL puromycin for 14–17 days.

### 4.3. RT-qPCR and RNA-Sequencing

RNA was isolated using TRIzolate (UD-Genomed URN0102) and total RNA was reverse transcribed using SuperScript III Reverse Transcriptase (ThermoFisher, Budapest, Hungary, 18064071). qPCRs were performed with SYBR Green Master Mix (Roche 4887352001) and gene-specific primers on a QuantStudio 12K Flex Real-Time PCR System (Applied Biosystems by ThermoFisher, Budapest, Hungary). Transcript levels were normalized to ACTB. Sequences of primers can be found in Table S1.

Library preparation and sequencing were performed using Illumina’s TruSeq RNA Sample Preparation version 2 by the Genomic Medicine and Bioinformatics Core Facility at the University of Debrecen, Debrecen, Hungary.

### 4.4. ChIP-Seq

20 million breast cancer cells (MCF-7 and T47D) were crosslinked with 1% methanol-free formaldehyde (Thermo Fisher Scientific, 28908) for 10 min at room temperature (RT). Formaldehyde was quenched using 0.125 M glycine for 5 min at RT and then cells were rinsed twice with ice-cold PBS. The cells were scraped up in 1 mL ChIP Lysis Buffer (1% Triton-X, 0.1% SDS, 150 mM NaCl, 1 mM EDTA, 20 mM Tris-HCl) and were centrifuged at high-speed. Nuclei were resuspended three times in ChIP Lysis Buffer. Sonication was performed in ChIP Lysis Buffer using a Bioruptor Plus Sonicator (Diagenode) 15 cycles (30 s on and 30 s off) at high intensity. After high-speed centrifugation the top 90% of sheared chromatin was diluted ten-fold with ChIP Lysis Buffer for immunoprecipitations. The following antibodies were used for immunoprecipitation overnight at 4 °C: ERα (sc-543X, 8 µg), NR2F2 (sc-271265X, 8 µg) and isotype control antibody (sc-2027 X, 8 µg). After centrifugation, the top 90% of supernatant were used for bead coupling. Antibody-chromatin-bead complex was incubated using pre-blocked Protein A-Protein G paramagnetic bead mix (1:1 ratio) (Thermo Fisher Scientific, cat. 10002D and 10004D) for 6 h at 4 °C. After the incubation, the captured beads were washed once with ChIP Wash Buffer 1 (1% Triton-X, 0.1% SDS, 150 mM NaCl, 1mM EDTA, 20 mM Tris-HCl, 0.1% NaDOC, Protease Inhibitor), twice with ChIP Wash Buffer 2 (1% Triton-X, 0.1% SDS, 500 mM NaCl, 1mM EDTA, 20 mM Tris-HCl, 0.1% NaDOC, Protease Inhibitor), once with ChIP Wash Buffer 3 (0.5% NP-40, 250 mM LiCl, 1mM EDTA, 20 mM Tris-HCl, 0.5% NaDOC, Protease Inhibitor) and twice with ChIP Wash Buffer 4 (200 mM Tris-HCl, 10 mM EDTA) using a magnetic rack. Antibody-chromatin-complex was eluted with 200 µL Bead Elution Buffer (1% SDS and 100 mM NaHCO_3_), and reverse crosslinking was carried out by adding 400 mM NaCl and incubating overnight at 65 °C. The samples were treated with 10 µg RNase and 20 µg Proteinase K. Immunoprecipitated DNA was purified using Qiagen’s MinElute PCR purification kit (cat. 28006).

Library preparation and sequencing were performed using Illumina’s TruSeq ChIP Sample Preparation by the Genomic Medicine and Bioinformatics Core Facility at the University of Debrecen, Debrecen, Hungary. Data is available in NCBI BioProject PRJNA602619.

Co-regulators and histones raw ChIP-seq data and processed ERα ChIA-PET data were downloaded from the Encyclopedia of DNA Elements (ENCODE) datasets [44,45] or Sequence Read Archive (SRA) datasets. Data is available with the following numbers: ENCSR752UOD (H3K27ac), ENCSR985MIB (H3K4me3), ENCSR493NBY (H3K4me1), ENCSR000EWS (GATA3), ENCSR000AHD (CTCF), GSM2137769 (FOXA1) and ENCSR000BZZ (processed ERα ChIA-PET).

### 4.5. Data Processing of ChIP-Seq and RNA-Seq

ChIP-Seq data were analyzed by a published computational pipeline [46]. Two biological replicates were merged and aligned to an hg19 reference dataset. Bam files were filtered out from duplicate reads and under quality number 20 using samtools [47]. Peaks were called using HMCan [13]. Artifact peak list was downloaded from the Encyclopedia of DNA Elements (ENCODE) and was removed from our peak sets.

Overlap regions were determined using diffBind, a Bioconductor package in R. Proportional Venn diagrams were visualized by BioVenn [48].

Motif enrichment analyses were performed using Homer software with findMotifsGenome.pl command. The size parameter was 100 bp. Tag density values were calculated based on summits of peaks flanking with ±500 base pair region for histograms and with ±1000 base pair region for heatmaps using Homer software with annotatePeaks.pl command options. Annotation was performed using Homer software with annotatePeaks.pl command.

Reads per kilobase per million mapped reads (RPKM) values were calculated on the summit ±50 bp region of the peaks. Regions of mother and daughter of ERα super-enhancers were determined by Bojcsuk et al. [22]. Loop numbers were evaluated using intersectBed between ERα or NR2F2 binding sites and anchor regions of ERα loops [49].

RNA-Seq data were aligned to an hg19 reference dataset using TopHat v2.1.1 [50]. Transcript abundances were calculated using featureCounts, and differentially expressed genes (FDR < 0.01, FC > |0.5|) were determined using edgeR [51,52]. Heatmaps were generated using the R pheatmap package. Gene Set Enrichment Analysis (GSEA) was performed to evaluate which gene sets were correlated with our differentially expressed genes with FDR < 0.25 using GSEA v3 software [53].

### 4.6. Data for Breast Cancer Patients

In case of breast cancer patients, mRNA z-scores data were derived from The Cancer Genome Atlas (TCGA) [54]. TCGA data were downloaded using the cBioPortal. Survival data were derived from KMPlotter [33].

### 4.7. Visualization

Heatmaps for ChIP-seq data were created using the Java TreeView software [55]. Plots were created using GraphPad prism software or R ggplot2.

### 4.8. Statistical Methods

Statistical analyses were performed with GraphPad prism software version 6. The Shapiro-Wilk test was used to test the normality distribution of data. Data were nonparametric distributed; we used the Mann-Whitney test. *p*-value significance was indicated with: * *p* < 0.05, ** *p* < 0.01, *** *p* < 0.001, **** *p* < 0.0001. For the Kaplan-Meier analysis, a Mantel-Cox test was used with significance at logrank *p* < 0.05.

## 5. Conclusions

In our paper, we demonstrated the presence of NR2F2 in regulation by ERα in breast cancer cells. Our results showed that high NR2F2 expression is correlated with better survival in luminal A breast cancer patients only. This result suggested the importance of NR2F2 in the ERα regulatory complex. We found high NR2F2 binding intensities with ERα co-bound by FOXA1 and GATA3 co-factors at active enhancer regions. NR2F2 overlaps with high-frequency ERα chromatin interactions within ERα-bound super-enhancers and plays a role in the expression of ERα target genes. Our results suggest that NR2F2 may play an important role in the survival and treatment of breast cancer patients with an ER-positive subtype.

## Supplementary Materials

Supplementary materials can be found at https://www.mdpi.com/1422-0067/21/6/1910/s1. Figure S1: COUP-TFII and ER binding sites in T47D cells. (**A**) Proportional Venn diagram shows the overlapping regions between ER and COUP-TFII. (**B**) IGV screenshot shows the ER and COUP-TFII binding sites at ER target genes in MCF-7 and T47D cells. (**C**) Histograms represent the ER and COUP-TFII tag density around shared and individual binding sites in T47D cells. Figure S2: IGV screenshot shows NR2F2 and ERα binding sites marked the mother and daughter regions of ERα SE, with ERα bound chromatin interactions at (**A**) JARID2 and (**B**) SIAH2 gene. Figure S3: mRNA level of COUP-TFII in MCF-7 and T47D followed by silencing using specific shRNA against COUP-TFII. Table S1: Sequences of primers.

## Abbreviations

NR2F2	Nuclear Receptor Subfamily 2 Group F Member 2
ERα	Estrogen receptor alpha
SE	Super-enhancer
