# Supplementary material for: NR2F2 Orphan Nuclear Receptor is Involved in Estrogen Receptor Alpha-Mediated Transcriptional Regulation in Luminal A Breast Cancer Cells
Source: Int J Mol Sci. 2020 Mar 11;21(6):1910. doi: 10.3390/ijms21061910 (PMC7139668; doi:10.3390/ijms21061910)
Supplement: Supplementary file 1 [file ijms-21-01910-s001.pdf]

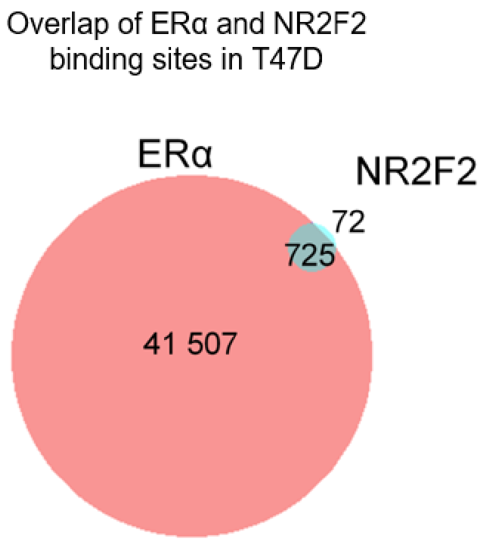

(A)

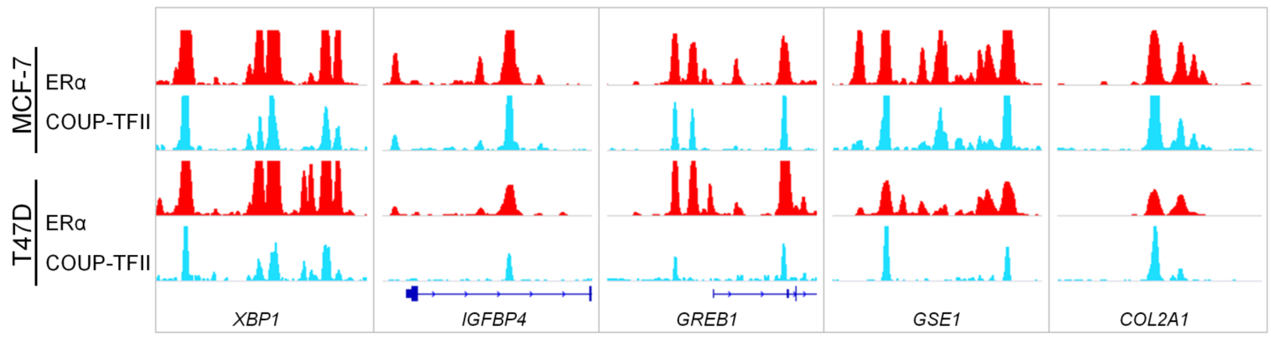

(B)

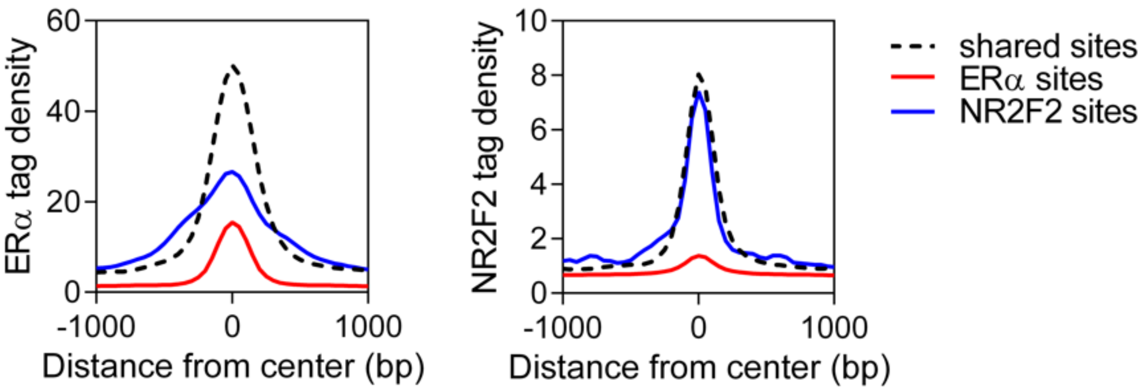

(C)

Figure S1. COUP-TFII and ER binding sites in T47D cells. **A**) Proportional Venn diagram shows the overlapping regions between ER and COUP-TFII. **B**) IGV screenshot shows the

ER and COUP-TFII binding sites at ER target genes in MCF-7 and T47D cells. **C**) Histograms represent the ER and COUP-TFII tag density around shared and individual binding sites in T47D cells.

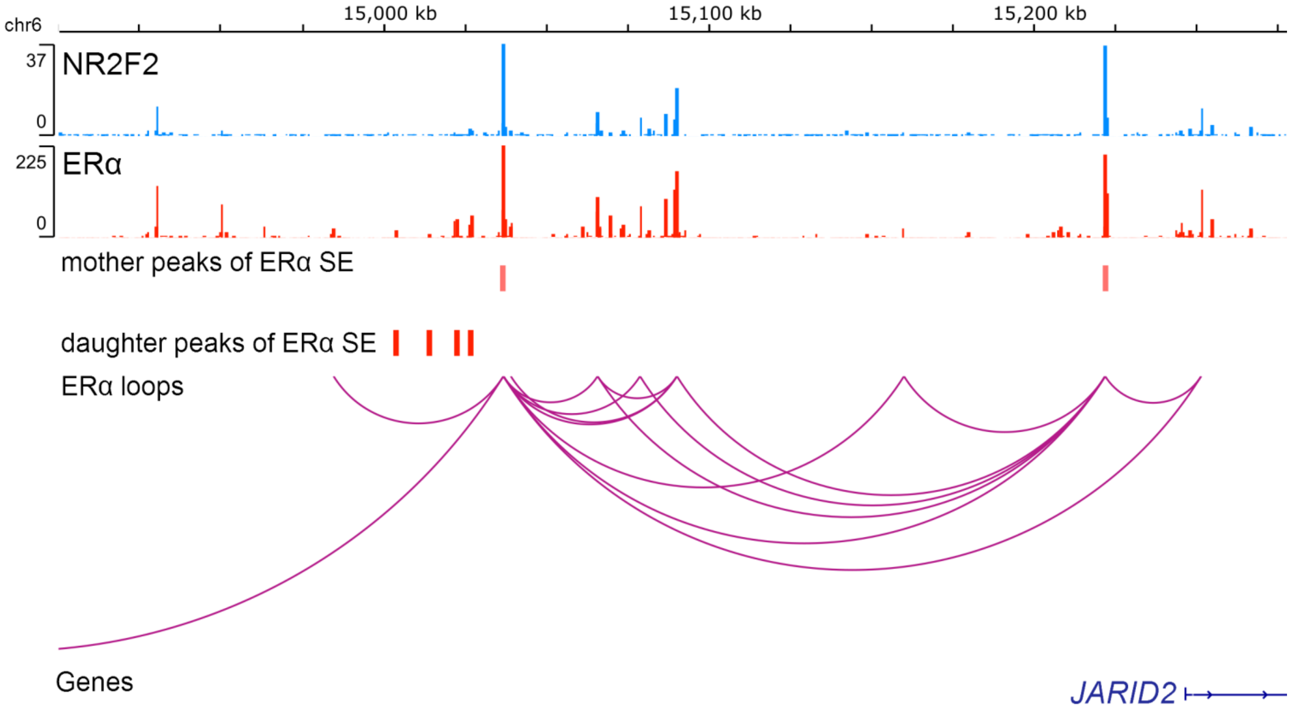

(A)

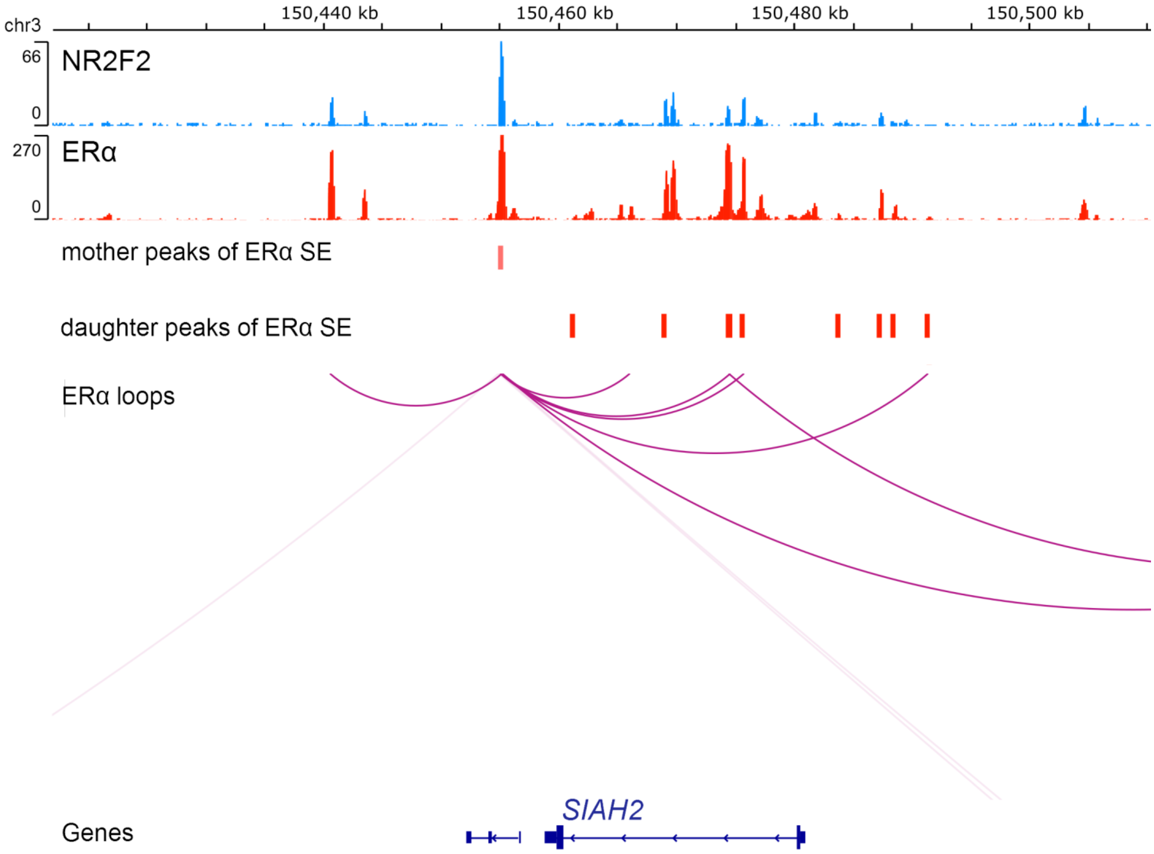

(B)

Figure S2. IGV screenshot shows NR2F2 and ER*α* binding sites marked the mother and daughter regions of ER*α* SE, with ER*α* bound chromatin interactions at **A**) JARID2 and **B**) SIAH2 gene.

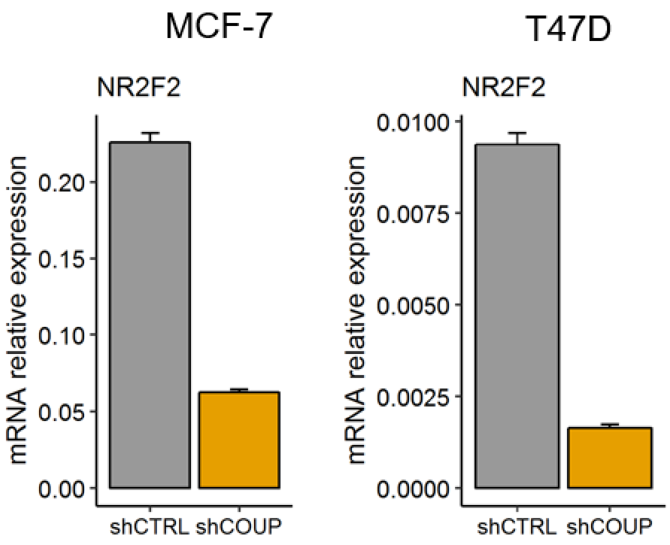

Figure S3. mRNA level of COUP-TFII in MCF-7 and T47D followed by silencing using specific shRNA against COUP-TFII.

Table S1. Sequences of primers.

Target Gene	Forward Primer	Reverse Primer
NR2F2	ccatagtcctgttcacctcaga	aatctcgtcggctggttg
BAMBI	ggctgcacgatgttctctc	cccggaaccacaactcttt
VEGFA	tgtgtgtgtgtgagtggttga	tctctgtgcctcgggaag
KRT15	ggtggtttcttcccacaaga	tgatgagagtggggagtgg
HEY2	atgagcataggattccgagagtg	ggcaggaggcacttctgaag
ACTB	ccctggcacccagcac	gccgatccacacggagtac
